# Relative contribution of three transporters to D-xylose uptake in *Aspergillus niger*

**DOI:** 10.3934/microbiol.2025037

**Published:** 2025-12-03

**Authors:** Jiali Meng, Astrid Müller, Jiajia Li, Vivien Bíró, Alexandra Márton, Erzsébet Fekete, Levente Karaffa, Miia R. Mäkelä, Ronald P. de Vries

**Affiliations:** 1 Fungal Physiology, Westerdijk Fungal Biodiversity Institute & Fungal Molecular Physiology, Utrecht University, Uppsalalaan 8, 3584 CT Utrecht, The Netherlands; 2 Department of Biochemical Engineering, Faculty of Science and Technology, University of Debrecen, Debrecen, Hungary; 3 Department of Bioproducts and Biosystems, School of Chemical Engineering, Aalto University, 02150 Espoo, Finland; # Current address: Ningbo Excare Pharm Inc., No.172, Xizishan Rd, Ningbo, Zhejiang, China

**Keywords:** Sugar transport, xylose, *Aspergillus niger*, gene expression, xylose uptake

## Abstract

The production of biofuels and chemicals from D-xylose is a promising option as D-xylose is the second most abundant sugar after D-glucose in lignocellulosic biomass. In microbes, efficient D-xylose uptake is a prerequisite for its utilization. Therefore, increasing D-xylose uptake efficiency by manipulation of D-xylose transporters would be an attractive strategy to improve fungal cell factories that use D-xylose as a substrate. In this study, we compared the contribution of three D-xylose transporters (XltA, XltB, XltD) from *Aspergillus niger* to overall D-xylose uptake at two D-xylose concentrations.

XltA and XltD contributed similarly to D-xylose uptake, while the role of XltB was minimal. However, even in the absence of all three transporters, D-xylose uptake still occurred, indicating the involvement of additional transporters. Surprisingly, there was no clear correlation between the kinetic characteristics of the transporters nor the expression profile of their corresponding genes with their influence on D-xylose transport. This suggests that selection of transporters for metabolic engineering of filamentous fungal cell factories based solely on kinetic parameters originating from heterologous expression of the transporters in yeast may not be a very efficient and reliable strategy.

## 1. Introduction

D-xylose is the second most abundant sugar after D-glucose in nature and a main component of hemicelluloses in lignocellulosic biomass, especially in xylan. Therefore, D-xylose is an attractive substrate for the production of biofuels and biochemicals [1]. Conversion of lignocellulosic biomass into biofuels and biochemicals by microorganisms includes the release of monomeric sugars (mostly D-glucose and D-xylose) from pretreated biomass and the microbial fermentation of sugars to the desired end products. Sugar uptake by microbial transporters is expected to be a rate determining step in this process. Therefore, increasing D-xylose uptake efficiency is likely to improve microbial fermentation and may be a promising strategy for developing robust microbial cell factories for D-xylose conversion [2]. High-affinity sugar transporters are expected to function at low sugar concentrations, while low-affinity sugar transporters would be more suitable for high sugar concentrations. Therefore, selection of sugar transporters for manipulation depends on the affinities of the transporters and sugar concentrations in the microbial fermentation.

Only a few native D-xylose transporters have been identified and characterized in fungi with highly diverse affinity for D-xylose (Table 1). These include two D-glucose/D-xylose transporters from the yeast *Candida intermedia* (Gfx1, GXS1) [3] (Table 1), one from *Candida tropicales* [4] and one from *Candida sojae* [5], while three D-glucose transporters of the yeast *Pichia stipitis* (Sut1, Sut2, Sut3) are also able to transport D-xylose, but with a considerably lower affinity than that observed for glucose [6]. Heterologous expression of Gxf1 and Sut1 in *Saccharomyces cerevisiae* led to significantly improved D-xylose utilization and ethanol production, respectively [7],[8]. Several transporters with D-xylose transport capacity from filamentous fungi have also been described (Table 1). In *Neurospora crassa*, three D-xylose transporters have been reported, An25 [9], and XAT-1 and XYT-1 [10]. XAT-1 can transport both D-xylose and L-arabinose, while XYT-1 can transport only D-xylose. XLT1 from *Trichoderma reesei* [11] is a high-affinity L-arabinose symporter with low affinity for D-xylose [12], while Xltr1 can transport D-xylose and D-glucose [13]. Another transporter, Str1, is involved in the utilization of diverse carbon sources in *T. reesei*, and is essential for pentose and pentitol utilization [14] despite having a higher affinity for D-glucose [15]. The Major Facilitator Superfamily (MFS) transporter XtrD from *Aspergillus nidulans* uses multiple sugars as a substrate, such as D-xylose, D-glucose, D-galactose, and D-mannose [16], but has high affinity for D-xylose, while a low-affinity D-glucose transporter, HxtB, has been shown to play a major role in D-xylose transport in this fungus [17],[18].

*Aspergillus niger* is a major fungal cell factory for the industrial production of organic acids, particularly citric acid, and industrially relevant enzymes [19]. Three candidate D-xylose transporters from *A. niger* (Table 1) were functionally validated and biochemically characterized in *S. cerevisiae* [15]. Of these, XltA can transport various sugars and showed a very high affinity for D-xylose, while XltB was suggested to be a specific low affinity D-xylose transporter. The affinity of XltC towards D-glucose was approx. 50 times higher than towards D-xylose, suggesting that its main function may be in D-glucose transport. Previous genomic and transcriptomic surveys of sugar transporters in *A. niger* revealed high diversity in the expression profiles of transporters with similar amino acid sequences, suggesting that they may either have a different function and/or their expression may be under control of different regulators [20],[21].

**Table 1.** Biochemically characterized sugar transporters involved in D-xylose utilization in fungi. MFS and ABC transporters represent the major facilitator superfamily and the ATP binding cassette transporters, respectively. The *K*_m_ for D-xylose of most transporters was determined in *S. cerevisiae*. The *A. niger* ortholog numbers are protein IDs from the *A. niger* NRRL3 genome [22] at Mycocosm [23]. ND = not determined.

Transporter	Species	*K*m (mM)		*A. niger* ortholog	Reference
		D-xylose	D-glucose		
Gxf1	*Candida intermedia*	ND	ND	5973	[3]
Gxs1	*C. intermedia*	0.4 ± 0.1	0.012 ± 0.004	3879	[3]
Sut1	*Scheffersomyces stipitis*	145.0 ± 1.0	1.5 ± 0.1	5973/8911/8621	[6]
Sut2	*S. stipitis*	49.0 ± 1.0	1.1 ± 0.1	5973/8911/8621	[6]
Sut3	*S. stipitis*	103.0 ± 3.0	0.8 ± 0.1	5973/8911/8621	[6]
Xyp29	*S. stipitis*	56.0 ± 9.4	ND	2351 (XltD)	[9]
An25	*Neurospora crassa*	175.7 ± 21.4	ND	235/935/817	[9]
XAT-1	*N. crassa*	18.17 ± 3.23	ND	2351 (XltD)	[10]
XYT-1	*N. crassa*	7.58 ± 0.60	ND	5973	[10]
XLT1	*Trichoderma reesei*	9.16 ± 3.35	ND	8663	[11],[12]
Str1	*T. reesei*	5.70 ± 0.19	0.01 ± 0.00	11715 (XltA)	[14],[15]
Str2	*T. reesei*	6.18 ± 0.81	0.05 ± 0.01	9364	[15]
Str3	*T. reesei*	2.19 ± 0.29	0.06 ± 0.01	8621	[15]
XtrD	*Aspergillus nidulans*	ND	ND	11715 (XltA)	[16]
HxtB	*A. nidulans*	ND	ND	3879	[17],[18]
XltA	*Aspergillus niger*	0.09 ± 0.03	0.07 ± 0.01	11715	[15]
XltB	*A. niger*	15.0 ± 4.50	ND	9716	[15]
XltC	*A. niger*	4.71 ± 1.04	0.11 ± 0.02	10052	[15]
MstA	*A. niger*	0.3 ± 0.1	0.025 ± 0.01	3147	[24]

It was shown that metabolic engineering of *A. niger* can generate strains that produce xylitol from wheat bran [25],[26], but overexpression of individual D-xylose transporter did not result in further increase in xylitol production [27]. To evaluate the contribution of individual D-xylose transporters on D-xylose uptake in *A. niger*, we selected three D-xylose transporters, XltA and XltB, and XltD (NRLL3_02351), the ortholog of XAT-1 from *N. crassa* (Table 1). XltC was not included due to its higher affinity for D-glucose, which may suggest this transporter is in fact a glucose transporter with side activity on xylose, similar to what was observed for *A. niger* MstA [24]. The genes encoding these transporters were deleted individually and in combination and the impact of the gene deletions on D-xylose uptake was assessed at two D-xylose concentrations.

## 2. Materials and methods

### 2.1. Strains, media, and growth conditions

*A. niger* strains used in this study are shown in Suppl. Table S1 and were deposited at the CBS culture collection of Westerdijk Fungal Biodiversity Institute. The uridine auxotrophic and non-homologous end-joining (NHEJ) deficient *A. niger* strain N593Δ*kusA* (CBS 138852) was used as the reference strain. CRISPR/Cas9 technology was used to create *A. niger* deletion mutants [28]. Details on the procedures used for this and the primers used for the creation of all deletion mutants are shown in Suppl. Data S1. *A. niger* protoplasting and transformation were carried out as described previously [29]. All *A. niger* strains were grown at 30 °C on Complete Medium (CM) or Minimal Medium (MM) [30] supplemented with required carbon source. For plate cultivations, 1.5% (w/v) agar was added, and 1.22 g/L uridine was supplemented for auxotrophic strains. A total of 1.3 mg/mL 5-fluoroorotic acid (5-FOA) was required in the solid plates for counter selecting colonies containing the *pyrG* marker gene on ANEp8-Cas9 plasmids.

*A. niger* strains were grown on CM plates with 1% D-glucose at 30 °C for 5 days. Conidia were harvested in 1.82 g/l N-(2-Acetamido)-2-aminoethanesulfonic acid (ACES) with 0.02% Tween 80 and were counted using a haemocytometer. Solid MM was used for growth profiles supplemented with required carbon sources, including 25 mM D-glucose, 2 mM D-xylose, 25 mM D-xylose, 1% (66.6 mM) D-xylose, 2 mM L-arabinose, 25 mM L-arabinose, and 1% (66.6 mM) L-arabinose. A total of 200 conidia in 5 µL ACES buffer were inoculated on the plates and incubated at 30 °C up to 9 days.

### 2.2. Sugar utilization rates

Pre-cultures were inoculated with 10^6^ conidia/mL and were grown for 16 hours with CM containing 2% D-fructose and 1.22 g/L uridine. Cultures were incubated at 30 °C in 500 mL Erlenmeyer flasks containing 100 mL aliquots in a rotary shaker at 250 rpm. Mycelia were then harvested by filtration on a sintered glass funnel without suction, washed with MM and transferred into fresh MM with 5 mM or 25 mM D-xylose, supplemented with 1.22 g/L uridine. The cultures were incubated in rotary shakers at 30 °C, 250 rpm, and samples were taken at several time points. The concentration of D-xylose in the culture medium was determined by HPLC analysis, using an H^+^ exchange column (Bio-Rad Aminex HPX-H^+^; Hercules, CA, USA), employing 10 mM H_2_SO_4_ at 55 °C as mobile phase. Compounds were detected by means of a refractive index detector [31]. Each point is the result of three averaged biological replicates, each performed as duplicate measurements, which deviated by no more than 5%.

### 2.3. Transcriptome analysis

Transcriptome analysis was performed using published data of *A. niger* grown on 25 mM of different monosaccharides [21]. Gene expression profiling was generated using the R package pheatmap (v1.0.10) based on normalized expression values (log₂ (FPKM +1)) [32]. Hierarchical clustering was applied to the genes, using the default distance metric and clustering method.

## 3. Results and discussion

### 3.1. Selection of D-xylose transporters

The biochemically characterized fungal sugar transporters show a high diversity in their affinity for D-xylose, based on previous studies (Table 1). However, it should be noted that the affinity of the majority of these transporters has been determined after heterologous expression in *S. cerevisiae*, and it has not been comprehensively established whether these affinities reflect the role of the transporters in the species of origin. To determine what the relative contribution of individual D-xylose transporters is to D-xylose uptake in *A. niger* we analyzed the biochemical properties and expression profiles of candidate D-xylose transporters of this species. Based on their published kinetic properties (Table 1), *A. niger* XltA and XltB have distinct affinities for D-xylose (0.09 and 15.0 mM, respectively) and these were therefore selected to determine the influence of a high (XltA) and medium-low (XltB) affinity transporter. *N. crassa* XAT-1 and *P. stipitus* Xyp29 are orthologs of *A. niger* XltD and have low affinity for D-xylose (18 and 56 mM, respectively) [9],[10]. We therefore assumed that XltD will have a similarly low affinity and therefore included this transporter in our study.

**Figure 1.**
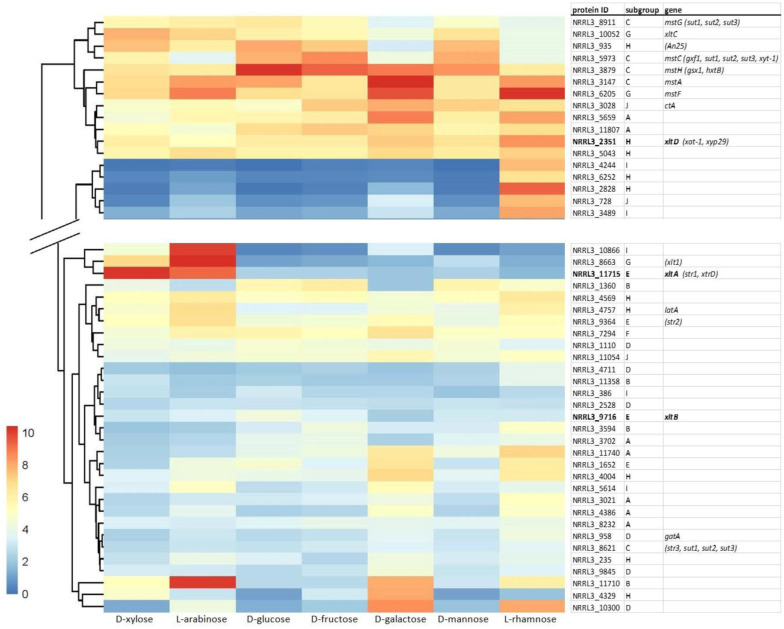
Expression profile of selected *A. niger* candidate sugar transporters. RNAseq analysis was performed on triplicate cultures of *A. niger* on 25 mM of the indicated sugars 2 h after transfer of the mycelium to this sugar. *A. niger* gene names are indicated in the right column, while gene names of orthologs in other fungi are in brackets. Transporters analyzed in this study are in bold. The grouping in the middle column is based on [21] and reflects putative functions. The color bar indicates the average log2 FPKM values. A = inositol/hexose, B = maltose/sucrose, C = hexose/pentose/polyol, D = uronic/quinic acid, E = xylose, F = unknown, G = pentose/hexose, H = glycerol/arabitol/pentose, I = unknown, and J = lactose/cellodextrin/xylobiose. A profile of all *A. niger* candidate transporters can be found in Figure S1.

The expression profile of these and other candidate sugar transporters [20] was re-analyzed using previously generated RNAseq data [21] of *A. niger* grown on 25 mM D-xylose, L-arabinose, D-glucose, D-fructose, D-galactose, D-mannose, and L-rhamnose (Figure 1, Figure S1). The choice for 25 mM was made based on it being high enough to not be limiting for growth but as low as possible to minimize the effect of carbon catabolite repression (unpublished data). Surprisingly, except for *xltA*, none of the (putative) D-xylose transporter encoding genes show specific expression during growth on D-xylose. This suggests a general lack of correlation between expression and function, except for some genes. In addition to the D-xylose specific expression of *xltA*, the ortholog of *T. reesei xlt1* shows specific expression on L-arabinose, which matches the L-arabinose transport specificity of Xlt1 [12]. Most of the other transporter encoding genes show either low expression on all tested substrates or expression on multiple substrates. However, two uncharacterized genes (NRRL3_11710, NRRL3_10866) show specific and high expression on L-arabinose, while one (NRRL3_2828) shows specific and high expression on L-rhamnose (Figure 1, Figure S1), making them promising candidates for L-arabinose and L-rhamnose transporters, respectively.

### 3.2. D-xylose utilization in *A. niger* involves multiple transporters

**Figure 2.**
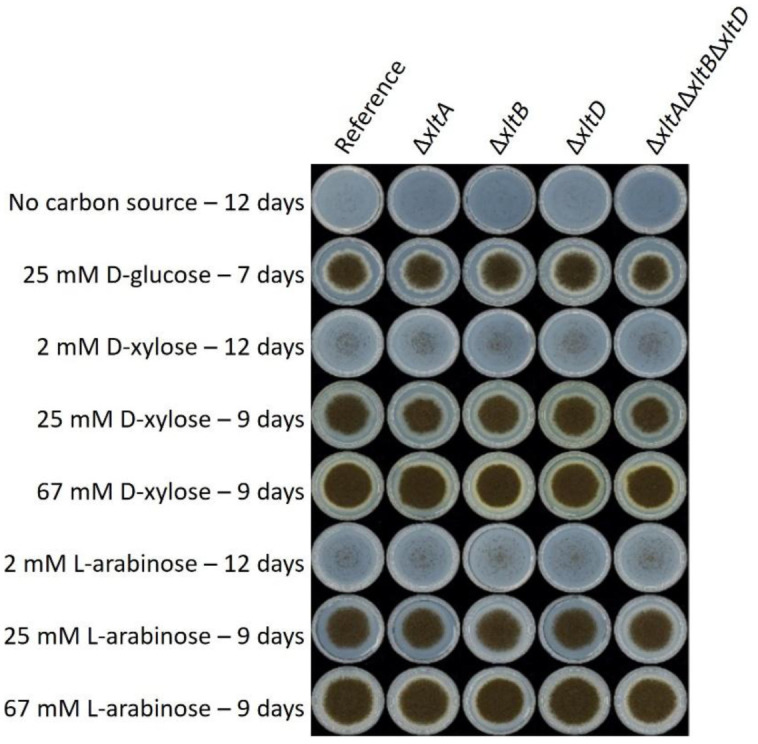
Growth profiling of the *A. niger* reference strain and deletion mutants of three D-xylose transporters, XltA, XltB, and XltD. The strains were grown in duplicate (without visual variation) on Minimal Medium [30] containing the indicated carbon sources at 30 °C for different times to maximize the chance of observing differences.

Single and triple deletion mutants of *xltA*, *xltB*, and *xltD* were generated in *A. niger*, and their growth was evaluated in duplicate on different concentrations of D-xylose and L-arabinose and compared to no carbon source and 25 mM D-glucose as controls (Figure 2). Growth at 2 mM D-xylose or L-arabinose was strongly reduced, suggesting that this carbon source cannot support growth of *A. niger*. In contrast, growth at 25 and 67 mM D-xylose and L-arabinose was similar in all strains and also similar to growth at 25 mM D-glucose. A very small reduction in growth is visible for the *xltA* and the triple mutant on 25 mM D-xylose, but overall, it can be concluded that on agar plates, the deletion of these transporters does not significantly affect the growth of *A. niger*. This indicates the involvement of other transporters contributing to D-xylose uptake. As indicated above, *A. niger* has several other candidate transporters that can (putatively) transport D-xylose, which apparently can compensate for the loss of XltA, XltB, and XltD.

**Figure 3.**
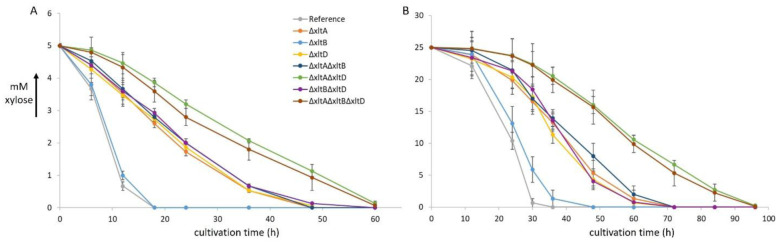
D-xylose utilization rates of the *A. niger* reference strain and deletion mutants of three D-xylose transporters, XltA, XltB, and XltD. The initial D-xylose concentrations are 5 mM (A) and 25 mM (B). The error bars represent standard deviations of biological triplicates.

Considering the expression of *xltA* on D-xylose and the confirmed function of XltA and XltB in D-xylose transport [15] as well as of the orthologs of XltD in *N. crassa* [10] and *P. stipitis* [9], the lack of a phenotype of the deletion strains was unexpected. To quantify their potential role in D-xylose transport, we performed D-xylose uptake assays of the single, double and triple deletion strains at two D-xylose concentrations, 5 mM and 25 mM. All strains were able to take up D-xylose at both concentrations, but uptake efficiency was affected by the deletion of the transporters (Figure 3). The strains split into three groups with respect to the D-xylose uptake profile. The *xltB* deletion strain behaved similarly to the reference strain and only shows slightly reduced D-xylose uptake at 25 mM (Figure 3B), suggesting that the contribution to D-xylose uptake of XltB is low under the tested conditions.

In contrast, strains in which *xltA* or *xltD* is deleted show more pronounced reduction in D-xylose transport (~2.7-fold and ~2.1-fold, respectively), compared to the reference strain at both D-xylose concentrations. Additional deletion of *xltB* together with *xltA* or *xltD* does not further reduce D-xylose uptake. However, even stronger reduction of D-xylose uptake was observed for a strain in which both *xltA* and *xltD* are deleted, irrespectively of whether *xltB* was deleted, indicating a cumulative effect of XltA and XltD. The uptake profiles at both D-xylose concentrations indicate that XltA and XltD contribute similarly to D-xylose transport. It is tempting to speculate that overexpression of XltA or XltD would result in increased D-xylose transport, and an aspect worth testing in future studies. However, overexpression of XltD in a xylitol accumulating mutant of *A. niger* did not increase xylitol accumulation, suggesting that D-xylose transport is not the limiting factor in that strain (unpublished data).

Surprisingly, the strong reduction in D-xylose uptake, as observed in liquid cultures (Figure 3), does not significantly affect growth on plates (Figure 2). Whether this means that D-xylose uptake occurs at higher rates than necessary in the reference strain or indicates a difference between solid and liquid cultures is not clear at this point. However, it has been shown that the cultivation setup affects gene expression and physiology of *A. niger* [33],[34].

Another unexpected result is that the kinetic parameters determined for these transporters in *S. cerevisiae* [15] do not seem to correlate with the reduction in D-xylose uptake observed in the deletion strains. The two concentrations used for the uptake assays, 5 mM and 25 mM, are in the range of the *K*_m_ of XltB and the characterized XltD orthologs, while the *K*_m_ for XltA is much lower (Table 1). However, XltB does not seem to contribute significantly to D-xylose uptake at these concentrations while the contribution of XltA and XltD are nearly identical. It cannot be excluded that the kinetic parameters of XltD differ from its orthologs (XAT-1 and Xyp29), but as these two transporters both have *K*_m_ values over 10 mM and the *K*_m_ of XltA is 0.09 mM (Table 1), it appears unlikely for XltD to have a *K*_m_ similar to that of XltA. In addition, high expression was observed for *xltA* at 25 mM D-xylose, while the expression of *xltD* is much lower (Figure 1). The very low expression of *xltB* on 25 mM D-xylose may, however, explain (in part) its minimal contribution to D-xylose uptake under these conditions.

The triple mutant shows considerable uptake of D-xylose at both concentrations, confirming the involvement of other transporters in overall D-xylose uptake. One candidate for this may be XltC, as this transporter was shown to transport D-xylose (although with much lower affinity than D-glucose) [15], and its corresponding gene shows expression on D-xylose as well as other carbon sources (Figure 1). Similarly, MstA was shown to transport D-xylose [24] and *mstA* is expressed on D-xylose (Figure 1). However, several other uncharacterized *A. niger* sugar transporter encoding genes show expression on D-xylose, suggesting an even broader set of transporters that may contribute to D-xylose transport. It would be relevant to identify and compare all transporters involved in D-xylose uptake in *A. niger* in future studies, similar to what was done in *S. cerevisiae* for D-glucose transport [35]. This could provide a D-xylose non-utilizing strain of *A. niger*, although the number of mutants that would need to be made and analyzed to compare their contribution to D-xylose transport in all gene deletion combinations would be extensive. We cannot exclude that deletion of *xltA* and/or *xltB* affected the expression of other candidate transporters that can transport D-xylose, so an evaluation of gene expression in these mutants would also be relevant in a future study.

## 4. Conclusions

In this study, we evaluated the contribution of three *A. niger* D-xylose transporters, XltA, XltB, and XltD, to overall D-xylose uptake. No clear correlation was observed between the transporters' (assumed) biochemical characteristics or the expression profiles of their corresponding genes and their physiological impact on D-xylose uptake in *A. niger*. XltA and XltD had a similar impact on D-xylose uptake, despite differences in expression profiles and (assumed) kinetic values, while XltB had only a minor impact. This may indicate that the determination of kinetic values by heterologous expression in *S. cerevisiae* may differ from their kinetic parameters in the original species or, alternatively, does not reflect their functional role in the original species. Care should therefore be given to selecting transporters for metabolic engineering of fungal cell factories based on such kinetic parameters. In addition, the specific expression of *xltA* on D-xylose, while *xltD* is expressed on a wider range of carbon sources, may suggest that XltD may function as a versatile sugar transporter that responds rapidly to various sugars, while XltA likely acts as a xylose-specific major transporter. This will need to be verified in future studies by analyzing the sugar specificity of XltD and by measuring uptake of other sugars XltD can transport in the Δ*xltD* strain.

## Use of AI tools declaration

The authors declare they have not used Artificial Intelligence (AI) tools in the creation of this article.





## References

[1] Lachke A (2002). Biofuel from D-xylose—The second most abundant sugar. Resonance.

[2] Kwak S, Jin YS (2017). Production of fuels and chemicals from xylose by engineered *Saccharomyces cerevisiae*: a review and perspective. Microb Cell Fact.

[3] Leandro MJ, Goncalves P, Spencer-Martins I (2006). Two glucose/xylose transporter genes from the yeast *Candida intermedia*: first molecular characterization of a yeast xylose-H^+^ symporter. Biochem J.

[4] Ribeiro Bueno JG, Borelli G, Ribeiro Corrêa TL (2020). Novel xylose transporter Cs4130 expands the sugar uptake repertoire *in recombinant Saccharomyces cerevisiae strains at high xylose concentrations*. Biotechnol Biofuels.

[5] Queiroz SS, Oliva B, Silva TF (2022). Integrated bioinformatics, modelling, and gene expression analysis of the putative pentose transporter from *Candida tropicalis* during xylose fermentation with and without glucose addition. Appl Microbiol Biotechnol.

[6] Weierstall T, Hollenberg CP, Boles E (1999). Cloning and characterization of three genes (SUT1-3) encoding glucose transporters of the yeast *Pichia stipitis*. Mol Microbiol.

[7] Katahira S, Ito M, Takema H (2008). Improvement of ethanol productivity during xylose and glucose co-fermentation by xylose-assimilating *S. cerevisiae* via expression of glucose transporter Sut1. Enzyme Microb Technol.

[8] Runquist D, Fonseca C, Radstrom P (2009). Expression of the Gxf1 transporter from *Candida intermedia* improves fermentation performance in recombinant xylose-utilizing *Saccharomyces cerevisiae*. Appl Microbiol Biotechnol.

[9] Du J, Li S, Zhao H (2010). Discovery and characterization of novel D-xylose-specific transporters from *Neurospora crassa* and *Pichia stipitis*. Mol Biosyst.

[10] Li J, Lin L, Li H (2014). Transcriptional comparison of the filamentous fungus *Neurospora crassa* growing on three major monosaccharides D-glucose, D-xylose and L-arabinose. Biotechnol Biofuels.

[11] Saloheimo A, Rauta J, Stasyk OV (2007). Xylose transport studies with xylose-utilizing *Saccharomyces cerevisiae* strains expressing heterologous and homologous permeases. Appl Microbiol Biotechnol.

[12] Havukainen S, Pujol-Gimenez J, Valkonen M (2021). Functional characterization of a highly specific L-arabinose transporter from *Trichoderma reesei*. Microb Cell Fact.

[13] Jiang Y, Shen Y, Gu L (2020). Identification and characterization of an efficient D-xylose transporter in *Saccharomyces cerevisiae*. J Agric Food Chem.

[14] Huang ZB, Chen XZ, Qin LN (2015). A novel major facilitator transporter TrSTR1 is essential for pentose utilization and involved in xylanase induction in *Trichoderma reesei*. Biochem Biophys Res Commun.

[15] Sloothaak J, Tamayo-Ramos JA, Odoni DI (2016). Identification and functional characterization of novel xylose transporters from the cell factories *Aspergillus niger* and *Trichoderma reesei*. Biotechnol Biofuels.

[16] Colabardini AC, Ries LN, Brown NA (2014). Functional characterization of a xylose transporter in *Aspergillus nidulans*. Biotechnol Biofuels.

[17] Dos Reis TF, de Lima PB, Parachin NS (2016). Identification and characterization of putative xylose and cellobiose transporters in *Aspergillus nidulans*. Biotechnol Biofuels.

[18] Dos Reis TF, Nitsche BM, de Lima PB (2017). The low affinity glucose transporter HxtB is also involved in glucose signalling and metabolism in *Aspergillus nidulans*. Sci Rep.

[19] Cairns TC, Nai C, Meyer V (2018). How a fungus shapes biotechnology: 100 years of *Aspergillus niger* research. Fungal Biol Biotechnol.

[20] Peng M, Aguilar-Pontes MV, de Vries RP (2018). *In silico* analysis of putative sugar transporter genes in *Aspergillus niger* using phylogeny and comparative transcriptomics. Front Microbiol.

[21] Xu L, Li J, Gonzalez Ramos VM (2024). Genome-wide prediction and transcriptome analysis of sugar transporters in four ascomycete fungi. Bioresour Technol.

[22] Aguilar-Pontes MV, Brandl J, McDonnell E (2018). The gold-standard genome of *Aspergillus niger* NRRL 3 enables a detailed view of the diversity of sugar catabolism in fungi. Stud Mycol.

[23] Grigoriev IV, Nikitin R, Haridas S (2014). MycoCosm portal: gearing up for 1000 fungal genomes. Nucleic Acids Res.

[24] vanKuyk PA, Diderich JA, MacCabe AP (2004). *Aspergillus niger mstA* encodes a high-affinity sugar/H^+^ symporter which is regulated in response to extracellular pH. Biochem J.

[25] Meng J, Chroumpi T, Mäkelä MR (2022). Xylitol production from plant biomass by *Aspergillus niger* through metabolic engineering. Bioresour Technol.

[26] Müller A, Meng J, Kuijpers R (2025). Exploring the complexity of xylitol production in the fungal cell factory *Aspergillus niger*. Enzyme Microb Technol.

[27] Meng J (2022). Fungal strain engineering: from understanding towards applications, PhD thesis, Department of Biology, Utrecht University, Utrecht, The Netherlands.

[28] Song L, Ouedraogo JP, Kolbusz M (2018). Efficient genome editing using tRNA promoter-driven CRISPR/Cas9 gRNA in *Aspergillus niger*. PLoS One.

[29] Kowalczyk JE, Lubbers RJM, Peng M (2017). Combinatorial control of gene expression in *Aspergillus niger* grown on sugar beet pectin. Sci Rep.

[30] de Vries R, Burgers K, van de Vondervoort P (2004). A new black *Aspergillus* species, *A. vadensis*, is a promising host for homologous and heterologous protein production. Appl Environ Microbiol.

[31] Fekete E, Karaffa L, Sandor E (2002). Regulation of formation of the intracellular β-galactosidase activity of *Aspergillus nidulans*. Arch Microbiol.

[32] Kolde R, Kolde MR (2015). Package ‘pheatmap’. R package.

[33] Garrigues S, Kun RS, Peng M (2021). The cultivation method affects the transcriptomic response of *Aspergillus niger* to growth on sugar beet pulp. Microbiol Spectr.

[34] Kun RS, Salazar-Cerezo S, Peng M (2023). The amylolytic regulator AmyR of *Aspergillus niger* is involved in sucrose and inulin Utilization in a culture-condition-dependent manner. J Fungi.

[35] Kruckeberg AL, Ye L, Berden JA (1999). Functional expression, quantification and cellular localization of the hxt2 hexose transporter of *Saccharomyces cerevisiae* tagged with the green fluorescent protein. Biochem J.

