# Supplementary material for: Relative contribution of three transporters to D-xylose uptake in Aspergillus niger
Source: AIMS Microbiol. 2025 Dec 3;11(4):877–88. doi: 10.3934/microbiol.2025037 (PMC12782949; doi:10.3934/microbiol.2025037)
Supplement: Supplementary file 1 [file microbiol-11-04-037-dataSI.pdf]

AIMS Microbiology

AIMS Microbiology, 11(4): 877–888.
DOI: 10.3934/microbiol.2025037
Received: 01 August 2025
Revised: 13 October 2025
Accepted: 17 October 2025
Published: 03 December 2025

https://www.aimspress.com/journal/microbiology

Research article

Relative contribution of three transporters to D-xylose uptake in *Aspergillus niger*

Jiali Meng[1,#], Astrid Müller[1], Jiajia Li[1], Vivien Bíró[2], Alexandra Márton[2], Erzsébet Fekete[2], Levente Karaffa[2], Miia R. Mäkelä[3] and Ronald P. de Vries[1,*]

[1] Fungal Physiology, Westerdijk Fungal Biodiversity Institute & Fungal Molecular Physiology, Utrecht University, Uppsalalaan 8, 3584 CT Utrecht, The Netherlands

[2] Department of Biochemical Engineering, Faculty of Science and Technology, University of Debrecen, Debrecen, Hungary

[3] Department of Bioproducts and Biosystems, School of Chemical Engineering, Aalto University, 02150 Espoo, Finland

[#]Current address: Ningbo Excare Pharm Inc., No.172, Xizishan Rd, Ningbo, Zhejiang, China

* **Correspondence:** Email: r.devries@wi.knaw.nl; Tel: +31302122600; Fax: +31302122601.

Supplemental Data S1: Construction of genetic mutants.

Table 1A. Plasmids and guide sequences used in this study.

	Description	CRISPR guide sequence	Reference
ANEp8-Cas9	Extra-chromosomal *cas9* expressing plasmid	-	Song *et al.* (2018)*
ANEp8-Cas9-gRNA (*xltA*)	ANEp8-Cas9 with gRNA targeting *xltA*	AATATTGTCGACCCAGAGCA	this study
ANEp8-Cas9-gRNA (*xltB*)	ANEp8-Cas9 with gRNA targeting *xltB*	ATTCTTGGAACGTTTCCCCG	this study
ANEp8-Cas9-gRNA (xltD; 02351)	ANEp8-Cas9 with gRNA targeting *xltD*	GCTGGTTCTCCAGCACAACG	this study

* Song, L., Ouedraogo, J. P., Kolbusz, M., Nguyen, T. T. M. and Tsang, A., 2018. Efficient genome editing using tRNA promoter-driven CRISPR/Cas9 gRNA in *Aspergillus niger*. PLoS One. 13, e0202868.

Table 1B. Primers used in this study. The 20 bp guide RNA (gRNA) sequences designed for the deletion of our target genes are indicated in red font. The linker sequence is depicted in lowercase/ bold font.

Primer name	Sequence (5' to 3')	Used for	Expected construct (bp)
xltA_F	TGCTCTGGGTCGACAATATTGACGAGCTTACTCGTTTCG	*construction of the gRNA (xltA)*	350
xltA_R	AATATTGTCGACCCAGAGCAGTTTTAGAGCTAGAAATAGCAAG	*construction of the gRNA (xltA)*	
left_xltA_F	GGCTGCTTTCATACCTTTCACG	*amplification of xltA 5' flank*	664
left_xltA_R	**cgatagcgaatcctagcagt**GGACAATCACAGTACAAACC	*amplification of xltA 5' flank*	
right_xltA_F	**actgctaggattcgctatcg**CGTCAGCAAAACGGTATAAGC	*amplification of xltA 3' flank*	587
right_xltA_R	GCCTCCCTCCACTGTAAGC	*amplification of xltA 3' flank/ checking the presence or absence of the xltA*	
nest_xltA_F	CCTACTACTTGATGTCATC	*fusion of xltA 5' and 3' flanks*	1187
nest_xltA_R	CGAGGTTTAGCGAGTAAC	*fusion of xltA 5' and 3' flanks*	
xltB_F	CGGGGAAACGTTCCAAGAATGACGAGCTTACTCGTTTCG	*construction of the gRNA (xltB)*	350
xltB_R	ATTCTTGGAACGTTTCCCCGGTTTTAGAGCTAGAAATAGCAAG	*construction of the gRNA (xltB)*	
left_xltB_F	GCAAGGTTAAGTCTTGATG	*amplification of xltB 5' flank*	541
left_xltB_R	**cgatagcgaatcctagcagt**CTTGGTCACACAGGCGATGC	*amplification of xltB 5' flank*	
right_xltB_F	**actgctaggattcgctatcg**GCATTGGTCTACTGGTAG	*amplification of xltB 3' flank*	659
right_xltB_R	CACTGTCAGAATATAACC	*amplification of xltB 3' flank/ checking the presence or absence of the xltB*	

Continued on next page

Primer name	Sequence (5’ to 3’)	Used for	Expected construct (bp)
nest_xltB_F	CGGCTTTCCATGTAGTTC	*fusion of xltB 5’ and 3’ flanks*	1047
nest_xltB_R	CTTGGCTTTCCGCTTCCGAG	*fusion of xltB 5’ and 3’ flanks*	
NRRL3_02351_F	CGTTGTGCTGGAGAACCAGCGACGAGCTTACTCGTTTCG	*construction of the gRNA (xltD)*	350
NRRL3_02351_R	GCTGGTTCTCCAGCACAACGGTTTTAGAGCTAGAAATAGCAAG	*construction of the gRNA (xltD)*	
left_02351_F	GAGATGTATCAGTCAGTC	*amplification of xltD 5’ flank*	711
left_02351_R	**cgatagcgaatcctagcagt**CGGACTTTCGCACTCTCTATG	*amplification of xltD 5’ flank*	
right_02351_F	**actgctaggattcgctatcg**GCGTACACGACTGTGCTTTC	*amplification of xltD 3’ flank*	760
right_02351_R	CCACGGACGAGAGGACTGC	*amplification of xltD 3’ flank/ checking the presence or absence of the xltD*	
nest_02351_F	CAGGCTTCTGTGGCTTAG	*fusion of xltD 5’ and 3’ flanks*	1350
nest_02351_R	CCTTGTGAGATATAGCAG	*fusion of xltD 5’ and 3’ flanks*	
linker_F	ACTGCTAGGATTCGCTATCG	checking the presence or absence of the target genes	

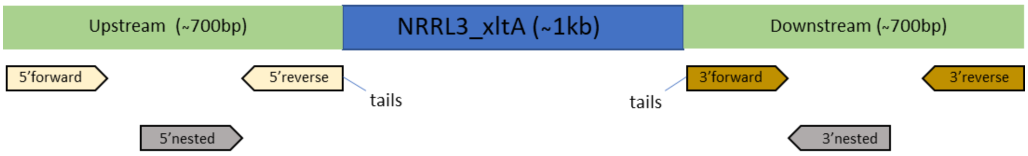

Figure 1. Schematic overview of rescue template assembly of transporter mutants.

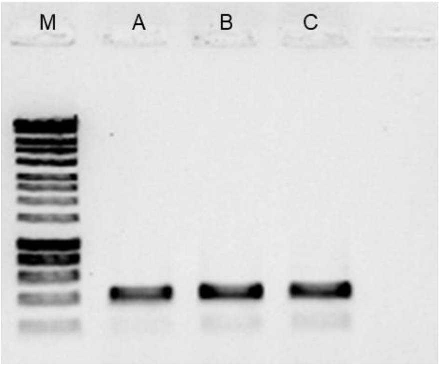

Figure 2A: PCR gel for gRNA cassettes for transporter mutants. A: *xltA*; B: *xltB*; C: *xltD*. Expected band size of 350bp could be confirmed.

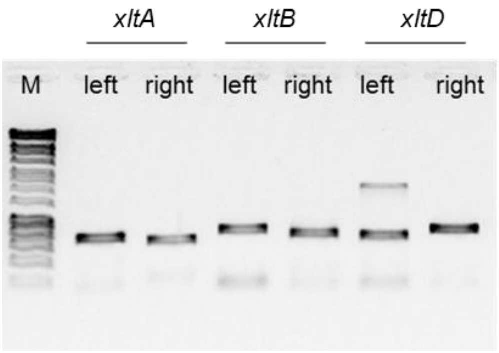

Figure 2B: PCR for 5' (left) and 3' (right) flanking regions of rescue templates of transporter mutants. PCR products were cut from gel and cleaned with Wizard SV **Gel** and PCR Clean-Up System (PROMEGA). For expected size see Table S1B.

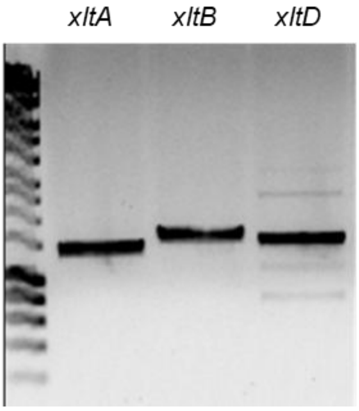

Figure 2C: Nested PCR to fuse 5' (left) and 3' (right) flanking regions of transporter mutants. PCR products were cut from gel and cleaned with Wizard SV Gel and PCR Clean-Up System (PROMEGA). For expected size see Table S1B.

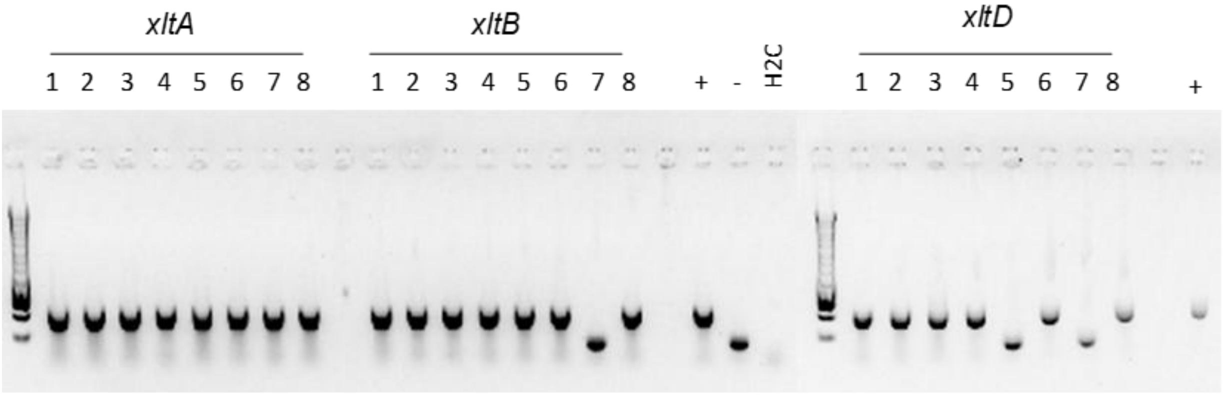

Figure 2D: *E. coli* DH5α transformation verification by PCR containing the ANEp8-Cas9-Lic transporter plasmids. Colony xltA-1, xltB-1 and xltD-4 were used for plasmid isolation.

Method 1: *A. niger* protoplasting and transformation protocol was based on Kowalcyk *et al.*, 2017:

Young mycelia from overnight culture were harvested by vacuum filtration, washed with 0.6 M $MgSO_4$ and dried between two sheets of paper. The mycelium was then dissolved in PS buffer (0.2 M sodium phosphate buffer, 0.8 M L-sorbitol, pH6) containing VinoTaste® Pro lysing enzyme (0.5 g enzyme/g of mycelia) and incubated in a rotary shaker at 100 rpm. When protoplasts were abundantly present, the mixture was filtrated through glass wool and undigested mycelia debris were removed. The protoplasts were collected by centrifugation (10 min, 1811 × *g*, 4 ℃), washed twice with ice-cold SC solution (182.2 g L^{-1} sorbitol, 7.35 g L^{-1} $CaCl_2$ * $2H_2O$) and resuspended in SC to a final concentration of 2 * 10^7 protoplast/mL. For transformation, 200 µL of fresh protoplast suspension, 20 µL of 0.4 M ATA (AurinTricarboxylic Acid ammonium salt), 100 µL of 20% PEG-4000 were mixed with 5 µg of deletion cassette DNA and incubated for 10 min. After addition of 1.5 mL 60% PEG-4000 the mixture was incubated for 20 min. Next, 5 mL 1.2 M sorbitol was added and incubated another 10 min. Transformed protoplasts were collected by centrifugation (10 min, 3220 × *g*), resuspended in 1 mL 1.2 M sorbitol and spread evenly over two selective plates using a cell spreader. Growing colonies were observed after 4 days. Putative deletion strains were purified by two consecutive single colony streaks. 1.3 mg/mL 5-fluoroorotic acid (5-FOA) was required for counter selecting colonies containing the pyrG marker gene on ANEp8-Cas9 plasmids. gDNA of 4-6 independent transformants per strain was isolated using standard phenol/chloroform extraction.

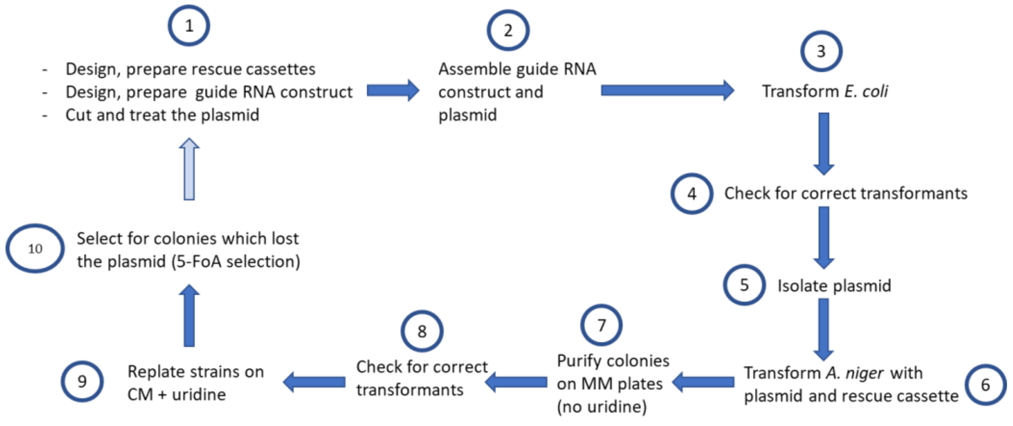

Figure 3A: Schematic workflow overview of construction and *A. niger* transformation.

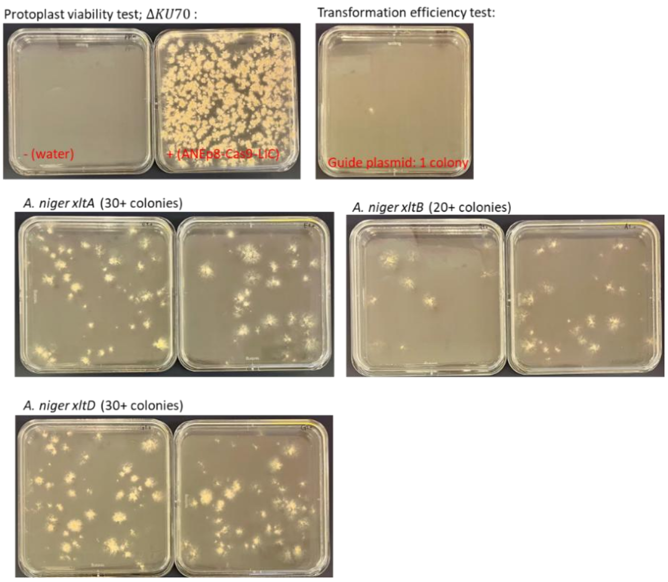

Figure 3B: Overview of transformation efficiency of transporter mutants. Tranformation of *A. niger xltA* yielded 30+ mutants, *xltB* yielded 20+ colonies and *xltD* yielded 30+ mutants. Putative deletion strains were purified by two consecutive single colony streaks and 5-fluoroorotic acid (5-FOA) was required for counter selecting colonies containing the pyrG marker gene on ANEp8-Cas9 plasmids.

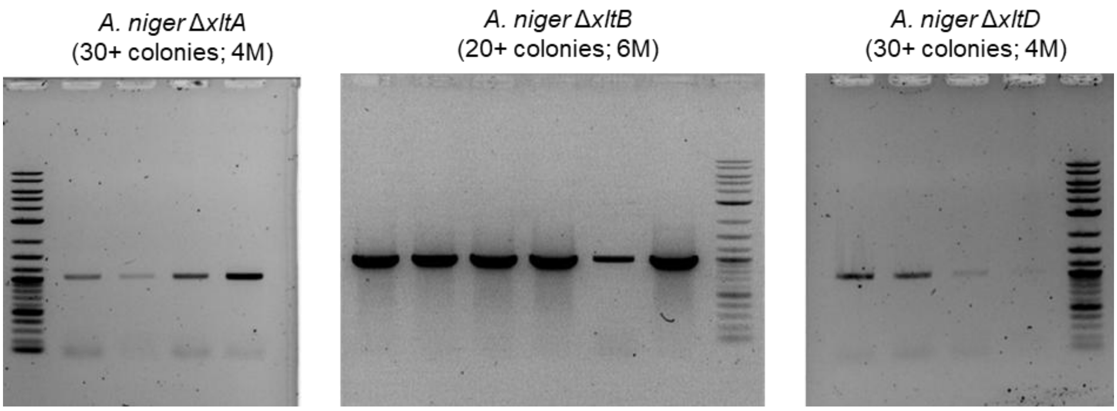

Figure 3C: PCR verification of transporter mutants. Mutant verification PCR conditions: left_xltA 5' Fw /right_ xltA 3' Rv, Program: 95 ℃ 5 min--> 35x (95 ℃ 1 min, 54 ℃ 1 min, 72 ℃ 4 min) --> 72 ℃ 10 min-->10 ℃ ∞; left_xltB 5' Fw /right_ xltB 3' Rv: Program: 95 ℃ 5 min--> 35x (95 ℃ 1 min, 52.4 ℃ 1 min, 72 ℃ 3 min) --> 72 ℃ 10 min-->10 ℃ ∞. Single integration verification: right_xltA 3'Rv/ right_ xltB 3' Rv/ right_ 02351 3' Rv + Linker F.

AIMS Press

© 2025 the Author(s), licensee AIMS Press. This is an open access article distributed under the terms of the Creative Commons Attribution License (https://creativecommons.org/licenses/by/4.0)
