# Supplementary material for: Relative contribution of three transporters to D-xylose uptake in Aspergillus niger
Source: AIMS Microbiol. 2025 Dec 3;11(4):877–88. doi: 10.3934/microbiol.2025037 (PMC12782949; doi:10.3934/microbiol.2025037)
Supplement: Supplementary file 2 [file microbiol-11-04-037-s001.pdf]

AIMS Microbiology

AIMS Microbiology, 11(4): 877–888.
DOI: 10.3934/microbiol.2025037
Received: 01 August 2025
Revised: 13 October 2025
Accepted: 17 October 2025
Published: 03 December 2025

https://www.aimspress.com/journal/microbiology

Research article

Relative contribution of three transporters to D-xylose uptake in *Aspergillus niger*

Jiali Meng[1,#], Astrid Müller[1], Jiajia Li[1], Vivien Bíró[2], Alexandra Márton[2], Erzsébet Fekete[2], Levente Karaffa[2], Miia R. Mäkelä[3] and Ronald P. de Vries[1,*]

[1] Fungal Physiology, Westerdijk Fungal Biodiversity Institute & Fungal Molecular Physiology, Utrecht University, Uppsalalaan 8, 3584 CT Utrecht, The Netherlands
[2] Department of Biochemical Engineering, Faculty of Science and Technology, University of Debrecen, Debrecen, Hungary
[3] Department of Bioproducts and Biosystems, School of Chemical Engineering, Aalto University, 02150 Espoo, Finland
[#]Current address: Ningbo Excare Pharm Inc., No.172, Xizishan Rd, Ningbo, Zhejiang, China

* **Correspondence:** Email: r.devries@wi.knaw.nl; Tel: +31302122600; Fax: +31302122601.

Supplementary

Table S1. *Aspergillus niger* strains used in this study.

Strain	CBS number	Genotype	Reference
N593Δ*ku70*	CBS 138852	*cspA1, kusA::amdS, pyrG*¯	Alazi et al, 2016
Δ*xltA*	CBS 147732	*cspA1, kusA::amdS, pyrG*¯, *xltA*¯	This study
Δ*xltB*	CBS 147733	*cspA1, kusA::amdS, pyrG*¯, *xltB*¯	This study
Δ*xltD*	CBS 147734	*cspA1, kusA::amdS, pyrG*¯, *xltD*¯	This study
Δ*xltA*Δ*xltB*Δ*xltD*	CBS 147741	*cspA1, kusA::amdS, pyrG*¯, *xltA*¯, *xltB*¯, *xltD*¯	This study

Reference: Alazi, E., et al., 2016. The transcriptional activator GaaR of Aspergillus niger is required for release and utilization of D-galacturonic acid from pectin. FEBS Lett. 590, 1804-1815.

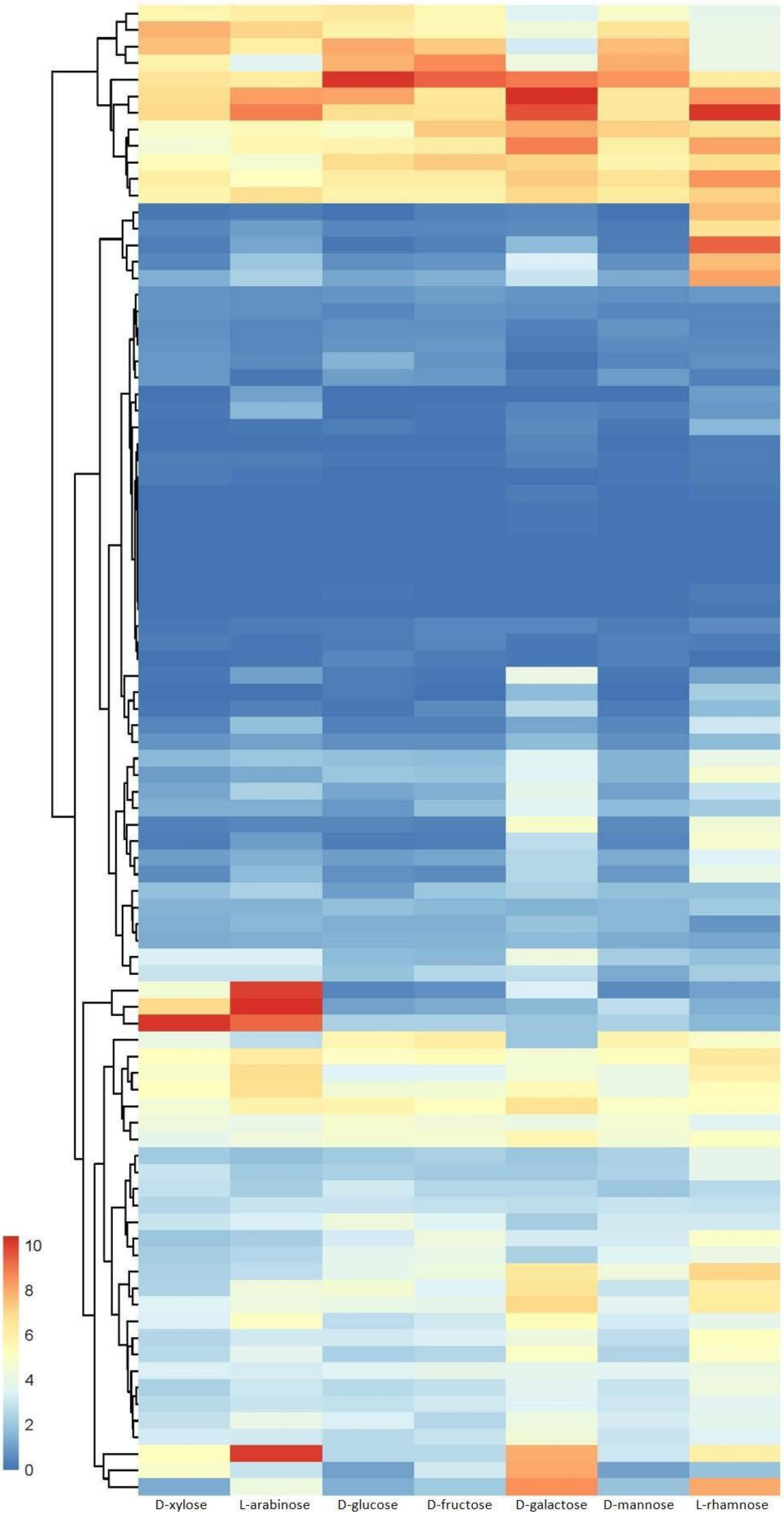

protein ID	subgroup	gene
NRRL3_8911	C	*mstG (sut1, sut2, sut3)*
NRRL3_10052	G	*xltC*
NRRL3_935	H	*(An25)*
NRRL3_5973	C	*mstC (gxf1, sut1, sut2, sut3, xyt-1)*
NRRL3_3879	C	*mstH (gsx1, hxtB)*
NRRL3_3147	C	*mstA*
NRRL3_6205	G	*mstF*
NRRL3_3028	J	*ctA*
NRRL3_5659	A	
NRRL3_11807	A	
**NRRL3_2351**	**H**	***xltD*** *(xat-1, xyp29)*
NRRL3_5043	H	
NRRL3_4244	I	
NRRL3_6252	H	
NRRL3_2828	H	
NRRL3_728	J	
NRRL3_3489	I	
NRRL3_6036	B	
NRRL3_5631	D	
NRRL3_11406	B	
NRRL3_11786	B	
NRRL3_6805	D	
NRRL3_7422	B	
NRRL3_9077	D	
NRRL3_10305	F	
NRRL3_5348	H	
NRRL3_1317	J	
NRRL3_10175	H	
NRRL3_1651	D	
NRRL3_815	A	
NRRL3_6866	D	
NRRL3_10164	H	
NRRL3_7442	E	
NRRL3_703	H	
NRRL3_6836	J	
NRRL3_3505	A	
NRRL3_7429	B	
NRRL3_1164	D	
NRRL3_1270	B	
NRRL3_5365	H	
NRRL3_3213	H	
NRRL3_3902	D	
NRRL3_11470	J	
NRRL3_7609	H	
NRRL3_8009	J	
NRRL3_8396	J	
NRRL3_8653	B	
NRRL3_10141	B	
NRRL3_11036	D	
NRRL3_3272	E	
NRRL3_10202	H	
NRRL3_8798	H	
NRRL3_8841	H	
NRRL3_11818	B	
NRRL3_5634	I	
NRRL3_834	B	
NRRL3_5552	H	
NRRL3_817	H	*(An25)*
NRRL3_4325	H	
NRRL3_10866	I	
NRRL3_8663	G	*(xlt1)*
**NRRL3_11715**	**E**	***xltA*** *(str1, xtrD)*
NRRL3_1360	B	
NRRL3_4569	H	
NRRL3_4757	H	*latA*
NRRL3_9364	E	*(str2)*
NRRL3_7294	F	
NRRL3_1110	D	
NRRL3_11054	J	
NRRL3_4711	D	
NRRL3_11358	B	
NRRL3_386	I	
NRRL3_2528	D	
**NRRL3_9716**	**E**	***xltB***
NRRL3_3594	B	
NRRL3_3702	A	
NRRL3_11740	A	
NRRL3_1652	E	
NRRL3_4004	H	
NRRL3_5614	I	
NRRL3_3021	A	
NRRL3_4386	A	
NRRL3_8232	A	
NRRL3_958	D	*gatA*
NRRL3_8621	C	*(str3, sut1, sut2, sut3)*
NRRL3_235	H	
NRRL3_9845	D	
NRRL3_11710	B	
NRRL3_4329	H	
NRRL3_10300	D	

Figure S1. Expression profile of *A. niger* candidate sugar transporters. RNAseq analysis was performed on triplicate cultures of *A. niger* on 25 mM of the indicated sugars. A. niger gene names are indicated in the right column, while gene names of orthologs in other fungi are in brackets. Transporters analyzed in this study are in bold. The grouping in the middle column is based on [18] and reflects putative functions. The color bar indicates the average log2 FPKM values. A = inositol/hexose, B = maltose/sucrose, C = hexose/pentose/polyol, D = uronic/quinic acid, E = xylose, F = unknown, G = pentose/hexose, H = glycerol/arabitol/pentose, I = unknown, J = lactose/cellodextrin/xylobiose.

AIMS Press

© 2025 the Author(s), licensee AIMS Press. This is an open access article distributed under the terms of the Creative Commons Attribution License (https://creativecommons.org/licenses/by/4.0)
